# Supplementary material for: Association of Diabetes Mellitus with All-Cause and Cause-Specific Mortality among Patients with Metabolic-Dysfunction-Associated Fatty Liver Disease: A Longitudinal Cohort Study
Source: J Pers Med. 2023 Mar 20;13(3):554. doi: 10.3390/jpm13030554 (PMC10057589; doi:10.3390/jpm13030554)
Supplement: Supplementary file 1 [file jpm-13-00554-s001.zip › jpm-2256680-supplementary.pdf]

**Supplementary Table S1. Serological parameters not penalized to zero by the LASSO model**

| serological parameters | regression coefficient |
|---|---|
| VitC | -0.002150863 |
| ALP | 0.001849605 |
| ALB | -0.018755530 |
| SCR | 0.443612383 |
| FIB-4 | 0.002219132 |
| NFS | 0.047963298 |

**Supplementary Table S2. Demographics and clinical characteristics in MAFLD patients, by diabetes status.**

| | Overall cohort (n=4,599) | Non-DM cohort (n=3,387) | DM cohort (n=1,172) | P value |
|---|---|---|---|---|
| Male, gender (%) | 2254 (49.4) | 1734 (51.2) | 520 (44.4) | < 0.001 |
| Age, yrs | 47 (35, 62) | 42 (32, 57) | 60 (48, 67) | < 0.001 |
| Ethnicity (%) | | | | 0.571 |
| Non-Hispanic white | 1579 (34.6) | 1189 (35.1) | 390 (33.3) | |
| Non-Hispanic black | 1088 (23.9) | 812 (24.0) | 276 (23.5) | |
| Mexican-American | 1716 (37.6) | 1256 (37.1) | 460 (39.2) | |
| Other | 176 (3.9) | 130 (3.8) | 46 (3.9) | |
| PIR (Poverty Income Ratio) | | | | < 0.001 |
| < 1 | 1099 (26.5) | 794 (25.6) | 305 (28.9) | |
| 1 ⩽ PIR < median | 990 (23.8) | 702 (22.7) | 288 (27.3) | |
| ⩾ median | 2063 (49.7) | 1602 (51.7) | 461 (43.7) | |
| Hypertension (%) | 1292 (28.4) | 819 (24.2) | 473 (40.4) | < 0.001 |
| BMI | | | | < 0.001 |
| < 25 | 830 (18.3) | 695 (20.6) | 135 (11.6) | |
| 25 ⩽ BMI < 30 | 1666 (36.7) | 1275 (37.7) | 391 (33.5) | |
| ⩾ 30 | 2049 (45.1) | 1408 (41.7) | 641 (54.9) | |
| ALT (U/L) | 18 (12, 27) | 18 (12, 27) | 18 (13, 27) | 0.273 |
| AST (U/L) | 21 (17, 27) | 21 (17, 27) | 20 (17, 27) | 0.082 |
| ALP (U/L) | 87 (72, 105) | 85 (71, 101) | 95 (77, 117) | < 0.001 |
| CRP (mg/dl) | 0.2 (0.2, 0.6) | 0.2 (0.2, 0.5) | 0.4 (0.2, 0.9) | < 0.001 |
| FRP (ng/mL) | 87 (72, 105) | 101 (47, 195) | 153 (77, 285) | < 0.001 |
| Vitamin A (umol/L) | 2.0 (1.6, 2.3) | 2.0 (1.6, 2.3) | 1.95 (1.6, 2.4) | 0.376 |
| Vitamin C (umol/L) | 35.8 (17.0, 52.2) | 35.8 (17.0, 52.8) | 35.8 (18.7, 51.7) | 0.981 |
| Vitamin E (umol/L) | 35.8 (17.0, 52.2) | 24.1 (19.6, 30.0) | 27.2 (21.6, 35.0) | < 0.001 |
| Serum selenium (nmol/L) | 1.6 (1.4, 1.7) | 1.6 (1.4,1.68) | 1.6 (1.5, 1.7) | < 0.001 |
| PLT (*10^9/L) | 270.5 (228.5, 320.0) | 272.0 (231.5, 321.0) | 263 (220, 315) | < 0.001 |

| | | | | |
|---|---|---|---|---|
| TC (mmol/L) | 1.7 (1.1, 2.5) | 1.6 (1.1, 2.3) | 2.1 (1.5, 3.1) | < 0.001 |
| TG (mmol/L) | 5.4 (4.7, 6.2) | 5.3 (4.6, 6.1) | 5.64 (4.91, 6.44) | < 0.001 |
| HDL-C (mmol/L) | 1.1 (1.0, 1.4) | 1.2 (1.0, 1.4) | 1.1 (0.9, 1.3) | < 0.001 |
| Total protein (g/L) | 74 (71, 77) | 74 (71, 77) | 75 (72,78) | 0.008 |
| Albumin (g/L) | 41 (39, 44) | 41 (39, 44) | 41 (38, 43) | < 0.001 |
| UA (umol/L) | 333.1 (273.6, 398.5) | 339.0 (279.6, 398.5) | 327.1 (261.7, 392.6) | 0.002 |
| Scr (mg/dl) | 1.0 (0.9, 1.2) | 1.0 (0.9, 1.2) | 1.0 (0.9, 1.2) | 0.158 |
| eGFR Category (%) | | | | < 0.001 |
| ⩾ 90(Stage 1 CKD) | 1092 (24.9) | 890 (27.4) | 202 (17.7) | |
| 60 ⩽ eGFR < 90(Stage 2 CKD) | 2600 (59.3) | 1944 (59.9) | 656 (57.6) | |
| 30 ⩽ eGFR < 60(Stage 3 CKD) | 670 (15.3) | 403 (12.4) | 267 (23.4) | |
| < 30(Stage 4-5 CKD) | 23 (0.5) | 9 (0.3) | 14 (1.2) | |
| FIB-4 Category (%) | | | | < 0.001 |
| < 1.3 | 3317 (76.3) | 2589 (80.5) | 728 (64.6) | |
| 1.3 ⩽ FIB-4 < 2.67 | 913 (21.0) | 564 (17.5) | 349 (31.0) | |
| ⩾ 2.67 | 115 (2.6) | 65 (2.0) | 50 (4.4) | |
| NFS Category (%) | | | | < 0.001 |
| < -1.455 | 2571 (58.6) | 2305 (70.9) | 266 (23.4) | |
| -1.455 ⩽ NFS < 0.676 | 1461 (33.3) | 818 (25.2) | 643 (56.5) | |
| ⩾ 0.676 | 357 (8.1) | 127 (3.9) | 230 (20.2) | |
| Severity of hepatic steatosis (%) | | | | < 0.001 |
| Mild | 1586 (34.8) | 1295 (38.2) | 291 (24.8) | |
| Moderate | 1987 (43.6) | 1432 (42.3) | 555 (47.4) | |
| Severe | 986 (21.6) | 660 (19.5) | 326 (27.8) | |

Continuous values are presented as medians (interquartile range) and categorical variables are presented as counts (percentages). DM, diabetes mellitus; Non-DM, patients without diabetes mellitus; BMI, body mass index; ALT, alanine transaminase; AST, aspartate transaminase; ALP, alkaline phosphatase; PLT, Serum selenium, platelet; TC, total cholesterol; TG, total triglyceride; HDL-C, high-density lipoprotein cholesterol; ALB, total protein, albumin; UA, uric acid; Scr, serum creatinine; eGFR, estimated glomerular filtration rate; NFS, NAFLD fibrosis score; FIB-4, Fibrosis-4 index.

**Supplementary Table S3. Cox survival analysis of risk factors for mortality in MAFLD patients**

| | univariate | | multivariate | |
|---|---|---|---|---|
| | HR, 95%CI | P value | HR, 95%CI | P value |
| age | 1.324 (1.191, 1.472) | < 0.001 | 1.071 (1.063, 1.079) | < 0.001 |
| gender | 1.077 (1.071, 1.083) | < 0.001 | 1.478 (1.297, 1.686) | < 0.001 |
| ethnicity | 0.819 (0.774, 0.868) | < 0.001 | 0.860 (0.801, 0.923) | < 0.001 |
| PIR | 0.892 (0.836, 0.952) | 0.001 | 0.796 (0.735, 0.860) | < 0.001 |
| hypertension | 1.936 (1.74, 2.154) | < 0.001 | 1.341 (1.190, 1.511) | < 0.001 |
| DM | 1.459 (1.31, 1.623) | < 0.001 | 1.416 (1.244, 1.611) | < 0.001 |
| BMI | 0.872 (0.81, 0.938) | < 0.001 | 0.841 (0.771, 0.917) | < 0.001 |
| VitC | 0.998 (0.995, 1.000) | 0.045 | 0.995 (0.992, 0.998) | < 0.001 |
| ALP | 0.997 (0.996, 0.998) | < 0.001 | 1.001 (1.000, 1.003) | 0.009 |
| ALB | 0.940 (0.926, 0.954) | < 0.001 | 0.949 (0.932, 0.967) | < 0.001 |
| SCR | 1.488 (1.404, 1.578) | < 0.001 | 1.303 (1.185, 1.433) | < 0.001 |
| NFS | 1.968 (1.817, 2.131) | < 0.001 | 1.315 (1.142, 1.513) | < 0.001 |

**Supplementary Figure S1. Association of DM with all-cause mortality in MAFLD of different (A) ages and (B) BMI.**

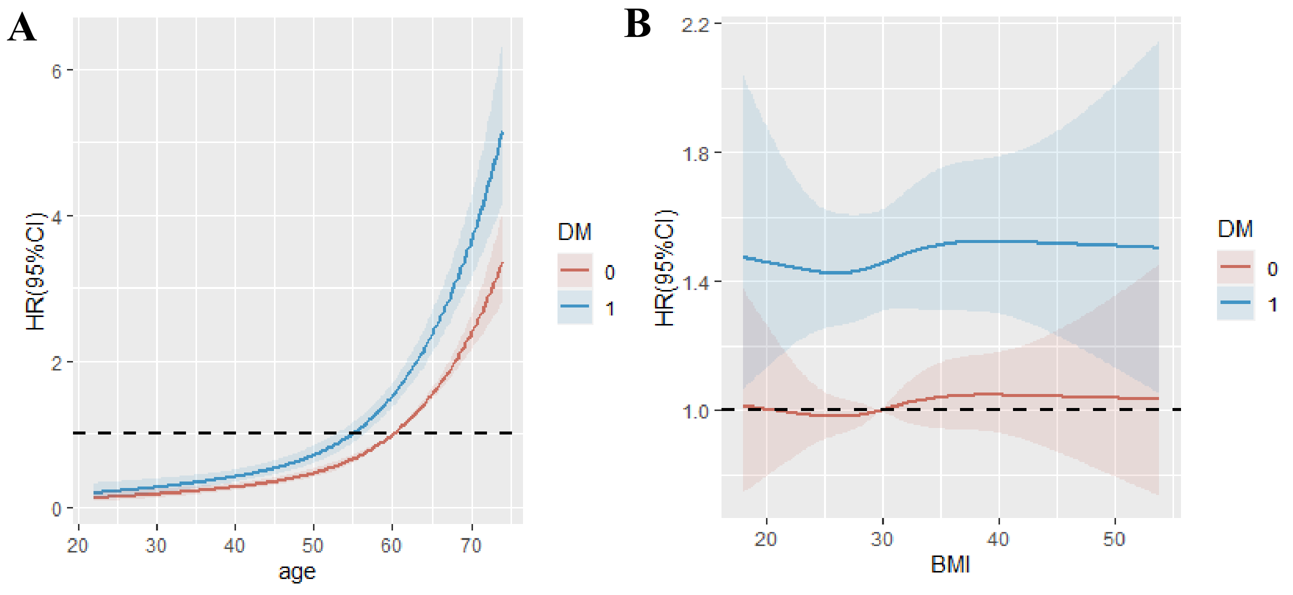

**Supplementary Figure S2. Subgroup analysis of the association of DM with cancer related mortality in MAFLD patients.**

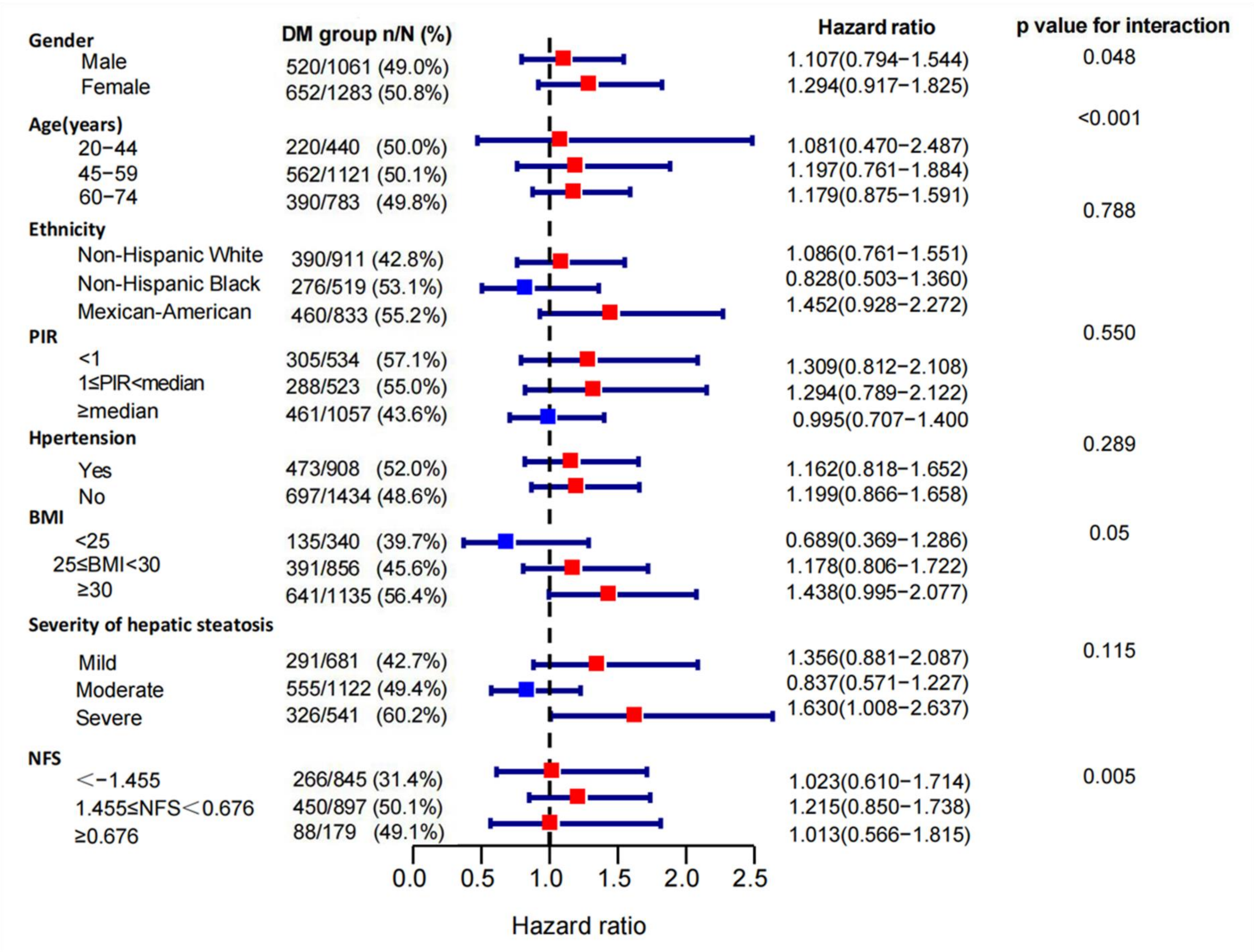

**Supplementary Figure S3. Subgroup analysis of the association of DM with non-cardiovascular related mortality in MAFLD patients.**

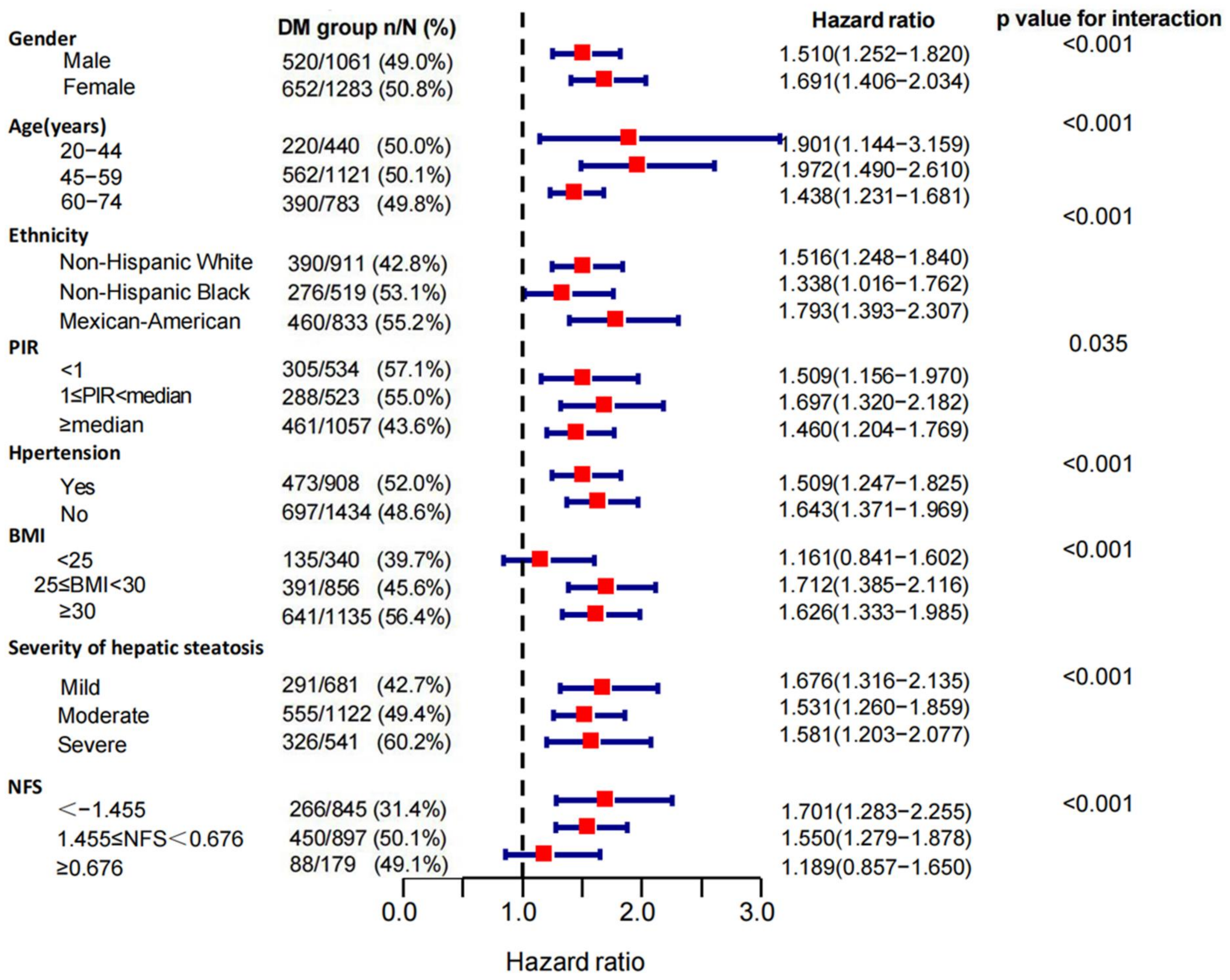

**FIGURE LEGENDS**

**Supplementary Figure S1. Association of DM with all-cause mortality in MAFLD of different (A) ages and (B) BMI.**

**Supplementary Figure S2. Subgroup analysis of the association of DM with cancer related mortality in MAFLD patients.**

Associations between DM and risk of cancer related mortality stratified by baseline age, gender, ethnicity, PIR (poverty income ratio), hypertension, BMI, severity of hepatic steatosis, and NFS scores in patients with MAFLD. The model was adjusted by age (not adjusted in subgroup analysis by age), gender (not adjusted in subgroup analysis by gender), ethnicity (not adjusted in subgroup analysis by ethnicity), PIR (not adjusted in subgroup analysis by PIR), hypertension (not adjusted in subgroup analysis by hypertension) and BMI (not adjusted in subgroup analysis by BMI). Diabetes, compared with non-diabetes patients.

**Supplementary Figure S3. Subgroup analysis of the association of DM with non-cardiovascular related mortality in MAFLD patients.**

Associations between DM and risk of non-cardiovascular related mortality stratified by baseline age, gender, ethnicity, PIR (poverty income ratio), hypertension, BMI, severity of hepatic steatosis, and NFS scores in patients with MAFLD. The model was adjusted by age (not adjusted in subgroup analysis by age), gender (not adjusted in subgroup analysis by gender), ethnicity (not adjusted in subgroup analysis by ethnicity), PIR (not adjusted in subgroup analysis by PIR), hypertension (not adjusted in subgroup analysis by hypertension) and BMI (not adjusted in subgroup analysis by BMI). Diabetes, compared with non-diabetes patients.
